# Aerosolized colistin for the treatment of nosocomial pneumonia due to multidrug-resistant Gram-negative bacteria in patients without cystic fibrosis

**DOI:** 10.1186/cc3020

**Published:** 2005-01-06

**Authors:** Argyris Michalopoulos, Sofia K Kasiakou, Zefi Mastora, Kostas Rellos, Anastasios M Kapaskelis, Matthew E Falagas

**Affiliations:** 1 Director, Intensive Care Unit, 'Henry Dunant' Hospital, Athens, Greece; 2 Research Fellow, Alfa HealthCare, Athens, Greece; 3 Attending Physician, Intensive Care Unit, 'Henry Dunant' Hospital, Athens, Greece; 4 Associate Director, Intensive Care Unit, 'Henry Dunant' Hospital, Athens, Greece; 5 Attending Physician, Alfa HealthCare and Department of Medicine, 'Henry Dunant' Hospital, Athens, Greece; 6 Adjunct Assistant Professor of Medicine, Tufts University School of Medicine, Boston, Massachusetts, USA and Director, Infectious Diseases Clinic, Department of Medicine 'Henry Dunant Hospital', Athens, Greece

**Keywords:** apnea, bronchoconstriction, colistin, inhaled, nosocomial pneumonia

## Abstract

**Introduction:**

The clinical and economic consequences of the emergence of multidrug-resistant Gram-negative bacteria in the intensive care unit (ICU) setting, combined with the high mortality rate among patients with nosocomial pneumonia, have stimulated a search for alternative therapeutic options to treat such infections. The use of adjunctive therapy with aerosolized colistin represents one of these. There is extensive experience with use of aerosolized colistin by patients with cystic fibrosis, but there is a lack of data regarding the use of aerosolized colistin in patients without cystic fibrosis.

**Methods:**

We conducted the present study to assess the safety and effectiveness of aerosolized colistin as an adjunct to intravenous antimicrobial therapy for treatment of Gram-negative nosocomial pneumonia. We retrospectively reviewed the medical records of patients hospitalized in a 450-bed tertiary care hospital during the period from October 2000 to January 2004, and who received aerosolized colistin as adjunctive therapy for multidrug-resistant pneumonia.

**Results:**

Eight patients received aerosolized colistin. All patients had been admitted to the ICU, with mean Acute Physiological and Chronic Health Evaluation II scores on the day of ICU admission and on day 1 of aerosolized colistin administration of 14.6 and 17.1, respectively. Six of the eight patients had ventilator-associated pneumonia. The responsible pathogens were *Acinetobacter baumannii *(in seven out of eight cases) and *Pseudomonas aeruginosa *(in one out of eight cases) strains. Half of the isolated pathogens were sensitive only to colistin. The daily dose of aerosolized colistin ranged from 1.5 to 6 million IU (divided into three or four doses), and the mean duration of administration was 10.5 days. Seven out of eight patients received concomitant intravenous treatment with colistin or other antimicrobial agents. The pneumonia was observed to respond to treatment in seven out of eight patients (four were cured and three improved [they were transferred to another facility]). One patient deteriorated and died from septic shock and multiple organ failure. Aerosolized colistin was well tolerated by all patients; no bronchoconstriction or chest tightness was reported.

**Conclusion:**

Aerosolized colistin may be a beneficial adjunctive treatment in the management of nosocomial pneumonia (ventilator associated or not) due to multidrug-resistant Gram-negative bacteria.

## Introduction

Nosocomial pneumonia due to multidrug-resistant Gram-negative bacteria, such as certain *Pseudomonas aeruginosa *and *Acinetobacter baumannii *strains, is among the most serious complications that occur in the intensive care unit (ICU) setting. Mortality, morbidity and health care costs are substantially increased by this type of infection [1-3]. Increasing rates of resistance among Gram-negative bacteria to most classes of antimicrobial agents have frequently led to clinical failure of currently employed therapies. Lack of development and introduction into clinical practice of new antibiotics to combat multiresistant Gram-negative bacteria have stimulated renewed interest in the use of the older antibiotic colistin.

Outcomes in patients with ventilator-associated pneumonia (VAP) due to multidrug-resistant Gram-negative bacteria are poor [1]. Intravenous colistin was recently used to treat such infections. Notably, a recent study [4] compared intravenous colistin (21 patients) with imipenem (14 patients) in the treatment of VAP due to multidrug-resistant *A baumannii*. Mortality rates were similar: 61.9% among patients treated with intravenous colistin and 64.2% among patients treated with imipenem. In patients with cystic fibrosis, aerosolized colistin has successfully been used to treat acute pulmonary exacerbations of infection or initial colonization with *P aeruginosa *strains [5,6]. However, there is a lack of data regarding the use of aerosolized colistin in patients without cystic fibrosis. A few reports have indicated that aerosolized colistin may be a beneficial additional therapeutic intervention in the management of nosocomial pneumonia (whether ventilator associated or not) [7-10]. In addition, a few old reports of the use of aerosolized polymyxin B yielded controversial results. Feeley and coworkers [11] reported that use of polymyxin B aerosol in seriously ill patients is associated with increased incidence of pneumonia due to polymyxin-resistant organisms. However, Klastersky and colleagues [12] found endotracheal administration of polymyxin B plus aminosidin to be a useful alternative regimen to endotracheal gentamicin for the prevention of lung infections.

We present data from our recent experience with aerosolized colistin for the treatment of pneumonia due to multidrug-resistant Gram-negative bacteria in eight ICU patients.

## Methods

### Design of the study and patient population

Patients who received colistin (Colomycin^®^, Forest Laboratories, Kent, UK, or Colistin^®^, Norma, Athens, Greece) for treatment of infections with multidrug-resistant Gram-negative bacteria from 1 October 2000 to 31 January 2004 at 'Henry Dunant' Hospital (a 450-bed tertiary care centre in Athens, Greece) were identified from the pharmacy electronic database. Medical records, specifically nursing records of medication administration, were retrospectively reviewed for all patients in order to identify those who received aerosolized colistin. One milligram of the colistin formulations used is approximately equal to 12,500 IU (Forest Laboratories, Kent) or 13,333 IU (Norma, Athens). Administration of aerosolized colistin for the treatment of nosocomial pneumonia due to Gram-negative bacteria, and review of patients' charts were approved by the institutional review board of the hospital.

### Data collection and entry

Data for several variables, including demographic and clinical information, as well as the results of laboratory and imaging tests (chest radiography or computed tomography of the thorax), were collected from the medical records of patients receiving aerosolized colistin. All available results of renal function tests (creatinine, urea, creatinine clearance, urinalysis), liver function tests (serum glutamate-pyruvate transaminase, serum glutamic-oxaloacetic transaminase, alkaline phosphatase, γ-glutamyltransferase, bilirubin), creatine phosphokinase and arterial blood gases were recorded during the course of colistin treatment and at hospital discharge.

### Microbiological testing

All causative micro-organisms were identified using routine microbiological methods. Susceptibility testing was done using both the disk diffusion method and an automated broth microdilution method (Vitek II; bioMerieux, Hazelwood, MO, USA). (The breakpoints were those defined by the National Committee for Clinical Laboratory Standards [13,14].) Susceptibility to colistin was tested by means of the disk diffusion method using a 10 μg colistin disk (Oxoid, Basingstoke, UK); isolates were considered sensitive if the inhibition zone was ≥ 11 mm. Intermediate sensitivity of isolated Gram-negative pathogens to antimicrobial agents was considered resistance. Multidrug-resistant was defined as resistance of the isolate to five antipseudomonal classes of antimicrobial agents (i.e. antipseudomonal penicillins, cephalosporins, carbapenems, monobactams, quinolones, colistin and aminoglycosides). An isolate was defined as colistin-only sensitive if it was resistant to all antipseudomonal agents except colistin.

### Definition of pneumonia

Diagnosis of pneumonia required two or more serial chest radiographs with at least one of the following: new or progressive and persistent infiltrate, consolidation, cavitation, or pleural effusion. In addition, patients were required to have had fever >38°C with no other recognized cause or an abnormal white blood cell count (leucopenia [<4000 white blood cells/mm^3^] or leucocytosis [≥ 12,000 white blood cells/mm^3^]), and at least two of the following: new onset of purulent sputum, change in the character of sputum, increased respiratory secretions, or increased requirement for suctioning; new onset or worsening of cough, or dyspnoea or tachypnoea; rales or bronchial breath sounds; or worsening gas exchange. Pneumonia was considered to be ventilator associated (VAP) when its onset occurred 48 hours after the initiation of mechanical ventilation, and was judged not to have been incubating before the initiation of mechanical ventilation [15].

### Definition of outcome

The definition of positive outcome (cure or improvement) of pneumonia was based on clinical (fever defervescence, resolution or partial resolution of presenting symptoms and signs of pneumonia, decrease in suctioning requirements), radiological (decrease or disappearance of presenting findings on chest x-ray), and laboratory findings (improvement in arterial blood gases, or normalization of white blood cell count and C-reactive protein).

## Results

From 1 October 2000 through 31 January 2004, 152 patients received treatment with intravenous colistin for infections with multidrug-resistant Gram-negative bacteria. Eight out of 152 patients were identified as having received aerosolized colistin for the management of Gram-negative nosocomial pneumonia. Table 1 describes the demographic and clinical features of these patients, including comorbidities, responsible pathogen(s) and susceptibility of the pathogen(s) to commonly tested antimicrobial agents, as well as the outcome of the infection and of the patient.

The mean age of the patients was 59.6 years and most of them were male (six out of eight). All patients had been admitted to the ICU, with a mean Acute Physiology and Chronic Health Evaluation II scores on the day of ICU admission and on day 1 of aerosolized colistin administration of 14.6 and 17.1, respectively. During the preceding 3 months, three patients had been hospitalized in the same or another unit. All patients had received other antimicrobial regimens before aerosolized colistin was initiated. In addition, three patients received immunosuppressive treatment (steroids) and four received immunoglobulin therapy during their hospitalization.

The responsible pathogens in the eight cases of nosocomial pneumonia were *Acinetobacter baumannii *(seven out of eight) and *P aeruginosa *(one out of eight) strains. Only in one case was a second strain isolated from the same culture specimen, and it was found to be methicillin-resistant *Staphylococcus aureus*. Half of the isolated pathogens were sensitive only to colistin; the rest were multidrug-resistant strains.

All patients received mechanical ventilatory support for a mean of 19.4 days. Colistin was prepared for nebulization; 1 or 2 million IU colistin was diluted in 2 or 4 ml sterile normal saline 0.9%, respectively. In patients undergoing mechanical ventilation aerosolized colistin was delivered by means of the Siemens Servo Ventilator 300 (Siemens-Elma AB, Solna, Sweden). In spontaneously breathing patients colistin was administered as follows: 1,000,000 IU were added to 4 ml normal saline and the solution was nebulized with 8 l/min oxygen flow and inhaled via a face mask. This technique of administration of aerosolized medication is commonly used worldwide for the administration of bronchodilators in nebulized form. The daily dose of aerosolized colistin ranged from 1.5 to 6 million IU divided into three or four doses, and the duration of administration ranged from 3 to 32 days (mean 10.5 days). No strictly uniform dosing strategy for aerosolized colistin was applied, and differences in regimen reflect the differing approaches of the individual attending physicians. In addition, seven out of eight patients received concomitant intravenous treatment with colistin or other antimicrobial agents with activity against Gram-negative bacteria, such as β lactams, quinolones and aminoglycosides. Only one patient received aerosolized colistin as monotherapy; she had received intravenous colistin therapy before aerosolized colistin for 7 days and continued to receive the intravenous therapy after the end of aerosolized therapy (for 32 days).

The pneumonia was observed to respond to treatment in seven out of eight patients who received supplemental therapy with aerosolized colistin. Four episodes of pneumonia were cured and three were improved at the end of treatment. Only one out of the eight patients who received aerosolized colistin for the treatment of multidrug-resistant Gram-negative pneumonia deteriorated and finally died. He was a 50-year-old multiple trauma patient, who was admitted to the ICU with fractures located at C4–C5, haemothorax and functional dissection of the spinal cord due to a car accident. His past medical history was noteworthy for arterial hypertension, Wolff–Parkinson–White syndrome, chronic renal insufficiency due to polycystic kidney disease and ankylosing spondylitis, for which he was receiving steroid therapy. During his prolonged hospitalization in the ICU, the patient developed pneumonia due to multidrug-resistant *A baumannii*, requiring intubation. His clinical condition became complicated by sepsis syndrome due to an infection caused by a colistin-only sensitive *P aeruginosa *strain, which was unresponsive to administered antimicrobial treatment. On day 95 of his hospitalization in the ICU, he died from septic shock and multiple organ failure.

Follow-up cultures were available for five out of eight patients. In four of them the responsible pathogen was eradicated, and in one case the pathogen persisted in repeated specimen cultures; this patient died. Superinfection with Gram-positive micro-organisms or yeasts was not observed. No Gram-negative bacterium developed resistance to colistin in subsequent specimen cultures during or at the end of aerosolized treatment.

Administration of aerosolized colistin was well tolerated by all patients. During treatment, all patients were closely monitored for possible respiratory adverse reactions, but none of them experienced chest tightness, bronchoconstriction, or apnoea. Only two patients, who had history of chronic obstructive pulmonary disease, received concurrent treatment with inhaled β_2 _agonist. Only in the patient who died did renal function worsen (baseline serum creatinine increased by 1.4 mg/dl) during aerosolized colistin treatment. This patient, as mentioned above, had a history of polycystic kidney disease and chronic renal failure, and died from septic shock and multiple organ failure. No deterioration in renal function was observed in the other seven patients during colistin treatment. One patient had baseline serum creatinine levels of 5.4 mg/dl, and at the end of colistin treatment serum creatinine had decreased to 4.5 mg/dl. That particular patient was already receiving haemodialysis treatment before the initiation of intravenous or aerosolized colistin.

Of 152 patients who received treatment with intravenous colistin for infections with multidrug-resistant Gram-negative bacteria during the period of study, 55 had received less than 72 hours of intravenous colistin and were excluded from all analyses. Medical records were not available for three patients; in addition, one patient was in the hospital during data collection. Thus, 93 patients were further analyzed. Forty-five of these patients received intravenous colistin for the treatment of nosocomial pneumonia due to Gram-negative bacteria. Survival and clinical cure rates for the infection were better, although not statistically significantly so, in patients with pneumonia who received additional aerosolized colistin than in patients who received only intravenous colistin treatment (survival: 7/8 patients [87.5%] versus 34/45 patients [75.6%], *P *= 0.41; clinical cure: 7/8 patients [87.5%] versus 30/45 patients [66.7%], *P *= 0.67).

## Discussion

Aerosolized colistin may be an effective adjunctive intervention for the treatment of nosocomial pneumonia due to multidrug-resistant Gram-negative bacteria in patients without cystic fibrosis. Colistin and polymyxin E are old antibiotics; colistin was almost abandoned for many years because of its reported nephrotoxicity and neurotoxicity. This medication was reintroduced into clinical practice just a few years ago, and this resulted mainly from increased resistance rates among Gram-negative bacteria, especially in the ICU setting, and the absence of new and effective alternative therapeutic options [16-18].

The idea of using colistin or polymyxin B (which belongs to the same group of antibiotics, and has similar antimicrobial spectrum, usage indications and toxicities as colistin) in the nebulized form for the management of pneumonia due to Gram-negative bacteria is not new. In 1963, Pino and coworkers [19] used aerosolized colistin in patients with pulmonary suppurations. A few years later, Marschke and Sarauw [20] reported two cases of pneumonia due to *P aeruginosa *strains in patients with underlying bronchiectasis and chronic bronchitis, in which polymyxin B was given by inhalation. Both patients experienced dyspnoea due to airway obstruction. Recently, aerosolized colistin was used successfully to treat and prevent pneumonia caused by *P aeruginosa *in patients with human immunodeficiency syndrome and in patients with nosocomial pneumonia and tracheobronchitis [21-23].

There is extensive experience with administration of aerosolized colistin to patients with cystic fibrosis, in whom this type of treatment is used to prevent or treat lung infections with *P aeruginosa *strains. Notably, studies found that nebulized colistin reduced the number of relapses of lung infections and subsequently the decline in lung function among patients with cystic fibrosis [24-27].

The pharmacokinetic properties and dosing strategies of aerosolized colistin are not well defined. Whether the various forms of colistin used for inhalation therapy (e.g. dry powder formulation for inhalation, colistin solutions for nebulization) or the different types of nebulizing systems influence the effectiveness and safety of colistin remains to be determined [28-31].

Adverse effects of aerosolized colistin or polymyxin B are a major concern; potential adverse effects include bronchoconstriction, chest tightness and apnoea due to neuromuscular blockade. One study conducted in 58 children with cystic fibrosis who received nebulized colistin for the treatment of lung infections [32] reported that 20 of them experienced a decrease in forced expiratory volume in 1 s by greater than 10% from baseline. In addition, another study [33] found that 35 out of 46 adult patients with cystic fibrosis who also received nebulized colistin for lung infection developed chest tightness. However, treatment with inhaled β_2 _agonists before the initiation of aerosolized colistin was able to prevent the development of such side effects in the respiratory system. Another significant concern regarding the use of aerosolized colistin for the treatment of nosocomial pneumonia is dissemination of multidrug-resistant bacteria through nebulizer devices [34,35]. However, this potential problem could be eliminated by strict use of appropriate infection control guidelines by medical and nursing hospital staff.

Our study is not without limitations. It is a small case series and is of a retrospective design. In addition, there is no control group of patients receiving treatment with only intravenous antimicrobial agents. Furthermore, some of the patients also received intravenous treatment with other antimicrobial agents, which might have influenced the outcomes.

Two major risks are arising from the wide use of colistin: the emergence of Gram-negative bacteria, such as *P aeruginosa *and *A baumannii*, resistant to colistin; and an increase of infections due to Gram-positive and Gram-negative pathogens, such as *Proteus *and *Serratia *spp., inherently resistant to colistin. Consequently, there is an urgent need to restrict the use of colistin use in order to minimize these risks.

## Conclusion

Inhaled colistin may be beneficial in the treatment of nosocomial pneumonia (ventilator associated or not) due to multidrug-resistant, Gram-negative bacteria. However, the severity of these infections in the ICU setting means that treatment just with aerosolized colistin is unlikely to be sufficient. This is in contrast to therapeutic strategies employed in patients with cystic fibrosis, in which initial lung colonization with *P aeruginosa *strains is commonly treated with aerosolized colistin alone. Randomized controlled trials studying the possible additional benefits and risks associated with use of nebulized colistin, as an adjunct to intravenous antimicrobial treatment, in patients with pneumonia due to multidrug-resistant Gram-negative bacteria are urgently needed.

## Key messages

• 	Aerosolized administration of colistin is a promising adjunctive therapy for management of patients with pneumonia (whether ventilator associated or not) due to multiresistant Gram-negative bacteria

• 	Aerosolized colistin was safe in this group of patients.

• 	There is an urgent need for randomized controlled trials examining the efficacy and safety of aerosolized colistin for the management of patients with nosocomial pneumonia.

## Abbreviations

ICU = intensive care unit; VAP = ventilator-associated pneumonia.

## Competing interests

The author(s) declare that they have no competing interests.

## Authors' contributions

AM and MEF conceived the study. SKK, ZM, KR and AMK collected data. All authors contributed to the writing and preparation of the manuscript.

## Figures and Tables

**Table 1:** Demographics, clinical features, responsible pathogens, and outcomes of patients treated with aerosolized colistin

Characteristic	Patient							
	
	1	2	3	4	5	6	7	8
Medical history	Fatty liver, arterial hypertension	Smoking, arterial hypertension, pulmonary oedema, heart attack, mild chronic renal failure	Liver hamartoma, chronic obstructive pulmonary disease, urinary incontinence, hypothyroidism, Sjögren's syndrome, excised left frontal lobe meningioma	Catarract, cholosteatoma, arterial hypertension, urinary tract infection 3 weeks before admission	Wolff–Parkinson–White syndrome, chronic renal failure (polycystic kidney disease), ankylosing spondylitis	Smoking, obesity, chronic obstructive pulmonary disease	Arterial hypertension, chronic renal dysfunction (creatinine clearance 75–80 ml/min), adenoma of hypophysis, epileptic seizures, cerebral haemorrhage	Arterial hypertension, cerebral arteriovenous malformation
Reason for admission	Stomach lymphoma	Acute myocardial infarction	Epileptic seizures	Fever, headache	Multitrauma patient, C4–C5 fractures due to car accident, functional dissection of spinal cord, haemothorax	Oesophageal perforation	Adenoma of hypophysis, cerebral haemorrhage	Pneumonia, sleep apnoea syndrome, cerebral haemorrhage
Discharge diagnosis	Stomach lymphoma, nosocomial pneumonia	Acute myocardial infarction, nosocomial pneumonia	Postsurgical intracranial haematoma, pulmonary embolism, inferior vena cava filter placement	Pneumococcal meningitis, hydrocephalus, pulmonary embolism, pneumonia, urinary tract infection	Septic shock, multiple organ failure	Mediastinitis	Pneumonia	Pneumonia, sleep apnoea syndrome, cerebral haemorrhage
APACHE II score on ICU admission	14	17	17	9	12	17	19	12
APACHE II score on first day of colistin treatment	10	29	19	8	19	20	18	14
Surgery during hospitalization	Liver biopsy, partial gastrectomy	Coronary artery bypass surgery	Drainage of postsurgical haematoma of left frontal lobe, inferior vena cava filter placement	Endoscopic ethmoidectomy, surgical drainage of the frontal and maxillary sinuses	Spinal arthrodesis surgery (C5–T1)	Surgical repair of oesophageal perforation	Excision of pituitary adenoma	Embolization of arteriovenous malformation
Duration of mechanical ventilation (days)	10	16	5	18	65	25	8	8
Time from ICU admission to develop the infection for which aerosolized colistin was given (days)	8	1	7	22	24	1	7	5
Site of infection	Pneumonia (VAP)	Pneumonia, urinary tract infection	Bacteraemia, pneumonia (VAP)	Pneumonia (VAP)	Pneumonia (VAP)	Pneumonia	Pneumonia (VAP)	Pneumonia (VAP)
Isolated micro-organism (source)	*Acinetobacter baumannii *(BAL)	*A baumannii *(bronchial secretions)	*A baumannii *(blood), *A baumannii *(bronchial secretions)	*Pseudomonas aeruginosa *(bronchial secretions)	*A baumannii *(bronchial secretions)	*A baumannii *(BAL)	*A baumannii *(bronchial secretions)	*A baumannii *(bronchial secretions)
Susceptibility of the isolated pathogen	MDR (sensitive to colistin and gentamycin)	COS	COS	COS	MDR (sensitive to colistin and gentamycin)	COS	MDR (sensitive to colistin and gentamycin)	MDR (sensitive to colistin and gentamycin)
Duration/dosage of nebulized colistin	6 days/1 million IU q8 h	13 days/1 million IU q8 h	10 days/0.5 million IU q8 h	5 days/1.5 million IU q8 h	7 days/2 million IU q8 h	3 days/1 million IU q8 h	8 days/0.5 million IU q6 h	19 days/1 million IU q8 h
Duration/dosage of concomitant intravenous antibiotic treatment	Colistin: 2 days/3 million IU q8 h, 6 days/2 million IU q8 h Levofloxacin: 3 days/500 mg q24 h Co-trimoxazole: 4 days/3 ampules q8 h Ciprofloxacin 4 days/400 mg q12 h	Colistin: 14 days/1 million IU q8 h Meropenem: 12 days/1 g q12 h	Colistin: 26 days/3 million IU q8 h Meropenem: 26 days/2 g q8 h	She received intravenous colistin before nebulized treatment (7 days/1 million IU q8 h) and after the end of nebulized treatment (32 days/1 million IU q8 h)	Tobramycin: 7 days/80 mg q24 h Aztreonam: 3 days/1 g q8 h	Colistin: 14 days/2 million IU q8 h Meropenem: 15 days/2 g q8 h Gentamicin: 8 days/80 mg q8 h	Colistin: 8 days/2 million IU q8 h Meropenem: 4 days/2 g q8 h	Meropenem: 27 days/2 g q8 h Gentamicin: 27 days/80 mg q8 h
Duration of hospitalization (days)	17	16	41	234	94	25	36	40
Duration of ICU stay (days)	11	16	21	62	95	25	13	20
Outcome of infection	Cure	Improvement	Cure	Improvement	Deterioration	Improvement	Cure	Cure
Outcome of patient	Discharge	Discharge	Discharge	Discharge	Death	Discharge	Discharge	Discharge
Serum creatinine value (mg/dl) on the first day of aerosolized colistin administration	1.1	5.2	1	0.4	2.4	0.6	0.8	0.8
Serum creatinine value (mg/dl) at the end of aerosolized colistin administration	0.8	4.5	0.9	0.5	3.8	0.5	0.7	0,6

APACHE, Acute Physiology and Chronic Health Evaluation; BAL, bronchoalveolar lavage; COS, colistin-only-sensitive; ICU, intensive care unit; MDR, multidrug-resistant; VAP, ventilator-associated pneumonia.
